# Oxidative Stress Response and Metal Transport in Roots of *Macleaya cordata* Exposed to Lead and Zinc

**DOI:** 10.3390/plants12030516

**Published:** 2023-01-23

**Authors:** Hongxiao Zhang, Xijing Sun, Delight Hwarari, Xinlong Du, Yinghao Wang, Huawei Xu, Shufang Lv, Ting Wang, Liming Yang, Dianyun Hou

**Affiliations:** 1 College of Agriculture, Henan University of Science and Technology, Luoyang 471023, China; 2 College of Biology and the Environment, Nanjing Forestry University, Nanjing 210037, China

**Keywords:** *Macleaya cordata*, histochemical staining, roots, lead, zinc, antioxidant

## Abstract

Heavy metal pollution possesses potential hazards to plant, animal and human health, which has become the focus of recent attention. Hence, phytoremediation has been regarded as one of the most important remediation technologies for heavy-metal-contaminated soils. In this research, a dominant mine tailing plant, *Macleaya cordata*, was used as the experimental material to compare the metal transport and oxidative stress response in its roots under lead (Pb) and zinc (Zn) treatments. The result showed that Pb was mainly accumulated in the roots of *M. cordata* under the Pb treatment; less than 1% Pb was transported to the parts above. An analysis of the Zn content demonstrated a 39% accumulation in the shoots. The production of reactive oxygen species was detected using the in situ histological staining of roots, which showed that hydrogen peroxide in the root tips was observed to increase with the increase in both Pb and Zn concentrations. No significant superoxide anion changes were noted in the root tips under the Pb treatment. An analysis of the root enzyme activity showed that increase in NADPH oxidase activity can be responsible for the production of superoxide anions, subsequent the inhibition of root growth and decrease in antioxidant enzyme activities in the roots of *M. cordata* exposed to excess Zn. In total, this research provides evidence that the root of *M. cordata* has a high antioxidant capacity for Pb stress, so it can accumulate more Pb without oxidative damage. On the other hand, the Zn accumulated in the roots of *M. cordata* causes oxidative damage to the root tips, which can stimulate more Zn transport to the shoots to reduce the damage to the roots. This result will provide a basis for the application of *M. cordata* in the phytoremediation of soil polluted by Pb-Zn compounds.

## 1. Introduction

With increase in mining activities as well as the use of pesticides and fertilizers on a large scale, heavy metal soil pollution has expanded rapidly. In particular, the heavy metal pollution of regional cultivated land has seriously affected the safety and quality of agricultural products [1]. In recent decades, phytoremediation had been widely used to alleviate heavy metal soil contamination, and it has attracted more and more attention due to its low cost, high efficiency, safety and environmental friendliness [1,2].

Plant tolerance to heavy metals is the primary consideration for phytoremediation technology [3]. Plants have evolved the mechanisms to tolerate and accumulate heavy metal in the process of adapting to their environment. Firstly, plants can control their heavy metal uptake and transport through the transporters located in their plasma membrane or they can sequester the heavy metals in vacuoles by tonoplast transporters [1,4]. Secondly, plants can induce the synthesis of specific organic compounds, including organic acids, amino acids, phytochelatins and metallothioneins, to directly bind with excess metal ions in cells or export them to the apoplast to act as barriers against heavy metals, thereby protecting the cell protoplasm [5]. Thirdly, the tolerance of most plants to heavy metals is significantly related to the activity of antioxidant enzymes [6,7,8], since heavy metals cause plant oxidative stress by replacing essential elements binding with proteins [9]. However, little is known on the tolerance mechanisms involved in the toxicity of lead (Pb) and excess zinc (Zn).

Pb is one of the most toxic environmental pollutants for plants, and Zn is an essential micronutrient associated with proteins and enzymes; however, excess Zn, like Pb, can interfere with numerous biochemical and physiological processes, including metal uptake and transport in plants [4]. The toxicities of Pb and excess Zn are mediated by the formation of reactive oxygen species (ROS), such as superoxide anions (O_2_^−^) and hydrogen peroxide (H_2_O_2_). ROS are toxic and can oxidize lipids, proteins and nucleic acids, thus causing lipid peroxidation, membrane damage, and enzyme inactivation [10,11]. Plants have antioxidant defense mechanisms that eliminate ROS, especially by enzymatic components such as superoxide dismutase (SOD), ascorbate peroxidase (APX), catalase (CAT), peroxidase (POD) and others. On the other hand, plasma-membrane-bound nicotinamide adenine dinucleotide phosphate (NADPH) oxidase is believed to be responsible for O_2_^−^ production in plants under heavy metal stress [10,11,12,13].

*Macleaya cordata*, a perennial medicinal plant in the family Papaveraceae, is a dominant plant with a large biomass in various mining areas, popular for its high tolerance and ability to accumulate various heavy metals; thus, it can be a good material for phytoremediation. Moreover, there are various bioactive alkaloids in this plant [14]; therefore *M. cordata* was approved by the European Food Safety Authority (EFSA) as a safe plant for the manufacture of feed additives [15]. Li et al. (2015) reported that *M. cordata* accumulated extremely high amounts of uranium, up to 278 times that of ordinary plants, and it can be used as a hyperaccumulator of uranium [16]. Wang et al. (2018) found that the molybdenum content of the aboveground parts of *M. cordata* reached 1207.4 mg·kg^−1^ in solution, and can be used as the hyperaccumulator of molybdenum [17]. Nie et al. (2016) also reported that *M. cordata* has a strong cadmium transport capacity [18]. In addition, the ability of *M. cordata* to successively accumulate lead, zinc and manganese has also been reported [19,20,21]. In a previous study, we investigated the accumulation of lead, zinc, cadmium and copper in *M. cordata* under hydroponic conditions, and found that *M. cordata* has a high tolerance and accumulation capacity for Pb and Zn [21]. This study aims to find out the different tolerance involved in oxidative stress and antioxidant protection in the roots of *M. cordata* exposed to Pb and excess Zn.

## 2. Results

### 2.1. Effects of Pb and Zn on Shoot Height and Root Length of M. cordata

**Figure 1:**
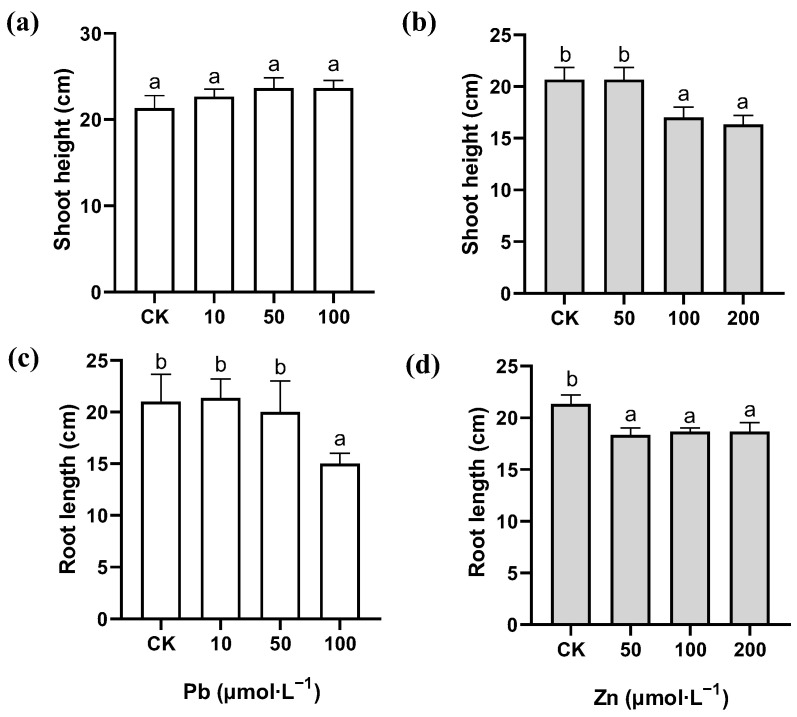
Shoot height (**a**,**b**) and root length (**c**,**d**) of *M. cordata* grown hydroponically under control (CK) and varied concentrations of Pb and Zn for seven days. Values are means ± SE (*n* = 3) with three separate replicates. Means denoted by different letters refer to the significant differences (*p* < 0.05, Duncan’s test).

The shoot height and root length of *M. cordata* was measured after seven days of the Pb and Zn treatments. Compared with that of the control, the shoot height of *M. cordata* was not significantly affected by the treatment except for 100 and 200 μmol·L^−1^ Zn concentrations (Figure 1a,b). However, 100 μmol·L^−1^ Pb and the three Zn concentrations significantly inhibited the elongation growth of the roots of *M. cordata* (Figure 1c,d).

### 2.2. Content of Pb and Zn in Roots of M. cordata

**Figure 2:**
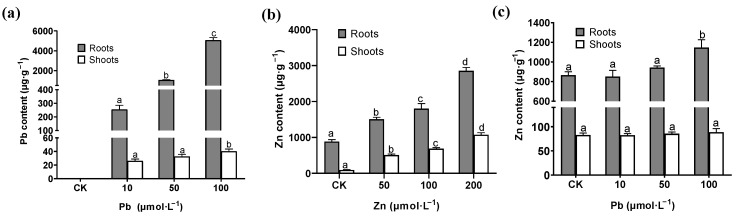
Pb (**a**) and Zn (**b**,**c**) content in shoots and roots of *M. cordata* grown hydroponically under control (CK) and varied concentrations of Pb and Zn for seven days. Values are means ± SE (*n* = 3) with three separate replicates. Means denoted by different letters refer to the significant differences (*p* < 0.05, Duncan’s test).

After seven days of the Pb and Zn treatments, the contents of Pb and Zn in the roots and shoots of *M. cordata* were detected, respectively. The results found that no Pb was detected in the control. Moreover, with the increase in the Pb treatment concentration, the Pb content in roots increased sharply, but the Pb content in shoots did not significantly increase until the treatment of 100 μmol·L^−1^ Pb, which was up to 0.7% of that in the roots (Figure 2a). This indicated that most of the Pb is retained in the *M. cordata* roots and is rarely transported to the plant’s aboveground parts. In contrast, the Zn content increased significantly in the roots and shoots of *M. cordata* under the Zn treatment, and the Zn content in the shoots was about 38% of that in the roots under the 100 and 200 μmol·L^−1^ Zn treatments (Figure 2b). This showed that the roots of *M. cordata* have a stronger ability to transport Zn to the shoots as compared to Pb. It is worth mentioning that the Zn content in the roots and shoots hardly changed under the Pb treatment, except when it significantly increased the under 100 μmol·L^−1^ Pb treatment (Figure 2c).

### 2.3. Effects of Pb and Zn on Production of ROS in Root Tips of M. cordata

**Figure 3:**
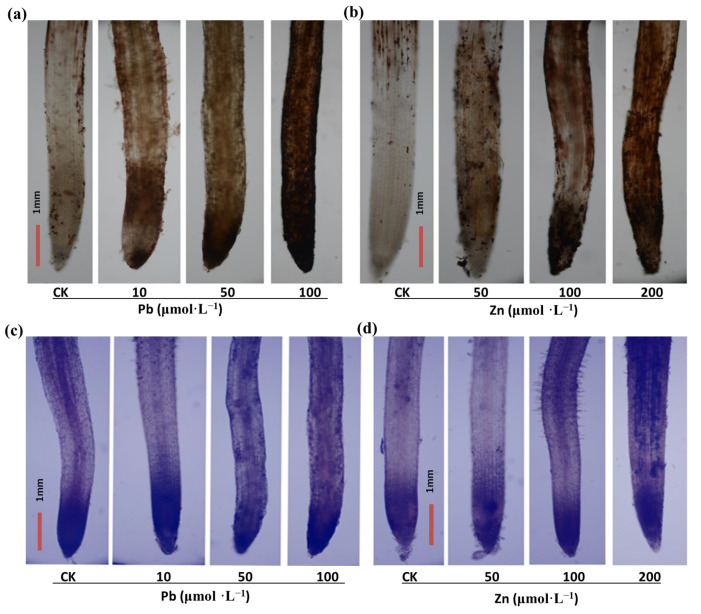
DAB and NBT staining of H_2_O_2_ (**a**,**b**) and O_2_^−^ (**c**,**d**) in roots of *M. cordata* grown hydroponically under control (CK) and varied concentrations of Pb and Zn for seven days. Bar, 1 mm. Staining experiments were repeated at least five times with similar results.

**Figure 4:**
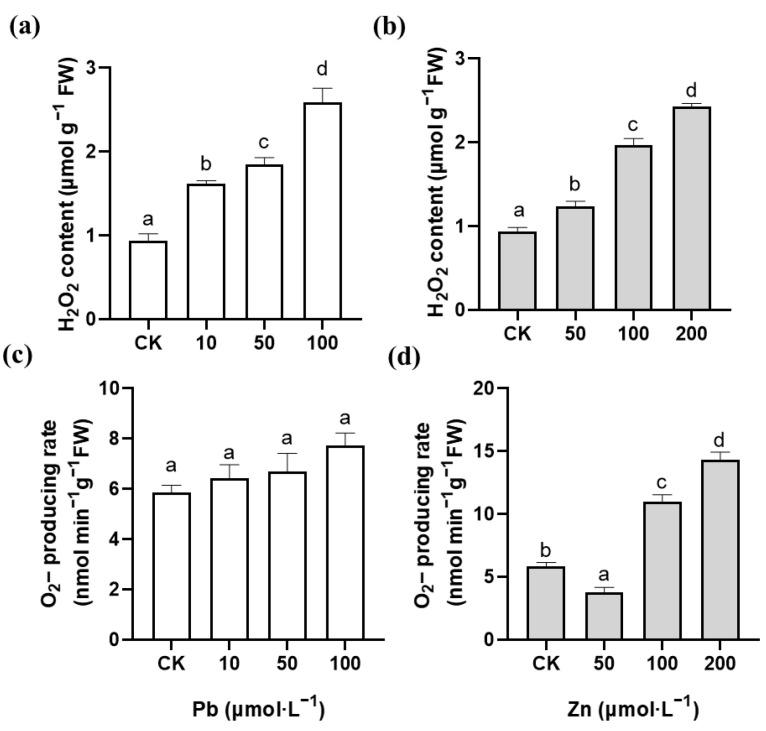
The determination of H_2_O_2_ content (**a**,**b**) and O_2_^−^-producing rate (**c**,**d**) in roots of *M. cordata* grown hydroponically under control (CK) and varied concentrations of Pb and Zn for seven days. Values are means ± SE (*n* = 3) with three separate replicates. Means denoted by different letters refer to the significant differences (*p* < 0.05, Duncan’s test).

3,3′-diaminobenzidine (DAB) staining in situ is based on the rapid reaction of H_2_O_2_ with DAB to form brownish-red spots. The amount of H_2_O_2_ in roots can be detected by observing the location of the colored spots. As shown in Figure 3a,b, the production of H_2_O_2_ in the root tips of *M. cordata* gradually increased with the increase of the Pb or Zn concentration, and a small amount of damage was observed on the root tips under the 200 μmol·L^−1^ Zn treatment. Nitroblue tetrazolium chloride (NBT) staining in situ relies on the reaction of O_2_^−^ with NBT to produce a blue product. Likewise, the amount of O_2_^−^ production in roots can be detected by observing the location of the blue spots. As shown in Figure 3c,d, blue-black precipitates appeared in the root tips of the control and treatment groups with different degrees of coloration. The O_2_^−^ production was not significantly increased in the root tips of the Pb treatment; however, the blue-black spots in the root tips were significantly increased under the 100 and 200 μmol·L^−1^ Zn treatments. Meanwhile, excess Zn, especially 100 μmol·L^−1^ Zn, evidently promoted root hair development. Furthermore, the H_2_O_2_ content and O_2_^−^-producing rate of the roots were basically consistent with the histochemical staining of the root tips of *M. cordata* (Figure 4).

### 2.4. Effects of Pb and Zn on Antioxidant Enzyme Activity in Roots of M. cordata

**Figure 5:**
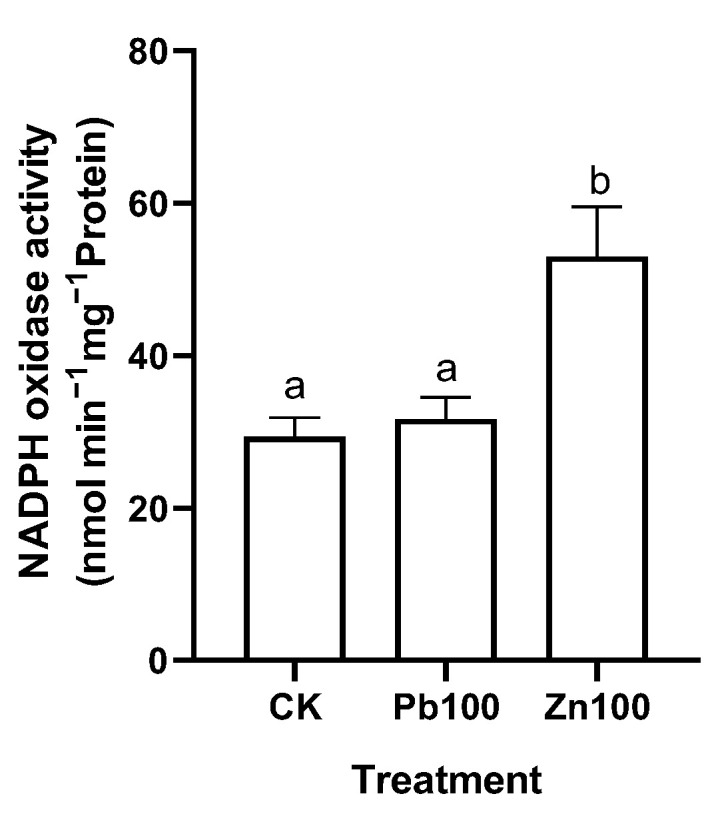
Activity of NADPH oxidase in roots of *M. cordata* grown hydroponically under control (CK), 100 μmol·L^−1^ Pb (Pb100) and Zn (Zn100) for seven days. Values are means ± SE (*n* = 3) with three separate replicates. Means denoted by different letters refer to the significant differences (*p* < 0.05, Duncan’s test).

**Figure 6:**
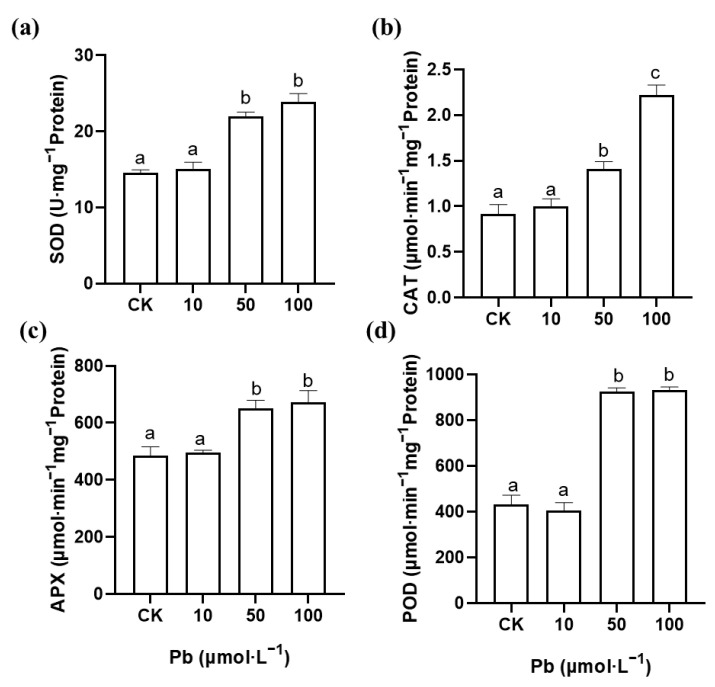
Activity of SOD (**a**), CAT (**b**), APX (**c**) and POD (**d**) in roots of *M. cordata* grown hydroponically under control (CK) and varied concentrations of Pb for seven days. Values are means ± SE (*n* = 3) with three separate replicates. Means denoted by different letters refer to the significant differences (*p* < 0.05, Duncan’s test).

**Figure 7:**
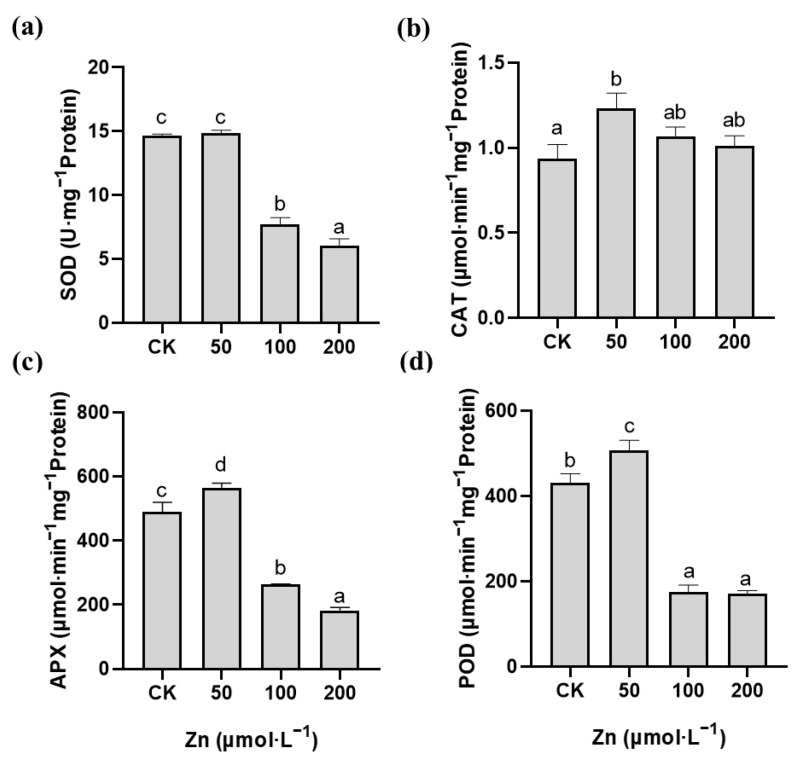
Activity of SOD (**a**), CAT (**b**), APX (**c**) and POD (**d**) in roots of *M. cordata* grown hydroponically under control (CK) and varied concentrations of Zn for seven days. Values are means ± SE (*n* = 3) with three separate replicates. Means denoted by different letters refer to the significant differences (*p* < 0.05, Duncan’s test).

In order to explore the source of the ROS in the *M. cordata* roots, the activity of the plasma membrane NADPH oxidase was analyzed. Compared with the control, the activity of NADPH oxidase in the roots of *M. cordata* significantly increased with 100 μmol·L^−1^ Zn, but not under the Pb treatment (Figure 5). Additionally, the enzyme activities of SOD, CAT, APX and POD significantly increased in the roots of *M. cordata* under 50 and 100 μmol·L^−1^ Pb; in particular, the POD activity increased more than twice than that of the control (Figure 6). In contrast, the roots exposed to the 100 and 200 μmol·L^−1^ Zn treatments had significantly lower enzyme activities of SOD, APX and POD compared to the control, while those in CAT, APX and POD showed an increase in activity with 50 μmol·L^−1^ Zn (Figure 7).

## 3. Discussion

When plants are subjected to heavy metal stress, the elongation growth of their roots is firstly inhibited, followed by their shoot height and dry weight [4]. In this research, the Pb treatment had no significant effect on the shoot height of *M. cordata*, while 100 and 200 μmol·L^−1^ Zn caused a significant decrease in the shoot height. These findings were consistent with those of a previous study on *M. cordata* treated with Pb and Zn for 20 d [21]. In addition, we showed that either 100 μmol·L^−1^ Pb or the three Zn concentrations significantly inhibit the elongation of a plant’s roots (Figure 1), showing *M. cordata* may have a stronger tolerance to Pb than Zn in the present study. Interestingly, the inhibition of root growth did not increase with a higher Zn concentration, which can be attributed to a good intracellular Zn homeostasis system in *M. cordata;* there was similar effect in the roots of *Elsholtzia haichowensis* under excess Cu [22].

One of the mechanisms of plant tolerance to heavy metals is to limit the transport of heavy metals to the aboveground parts or to the cells [5]. Our findings demonstrated that the amount of Pb accumulated in the roots of *M. cordata* was very high, reaching 5000 μg·g^−1^, but the transport ability to the shoots was less than 1% (Figure 2a). With the same concentration of Zn, the Zn content in the roots of *M. cordata* was lower than that of Pb, but the transport ability to the shoots was up to 39% (Figure 2b). In a previous study, when the treatment was increased to 1000 μmol·L^−1^ Pb and Zn for 20 d, the Zn content of the roots of *M. cordata* exceeded that of Pb [21], which can be attributed to the serious damage of the roots caused by the high concentration Pb. Pb could easily form Pb-binding compounds with cysteine, non-protein thiols, which caused Pb deposition in vacuoles or cell walls, and decreased the Pb transport in the plants [23]. It was reported that the levels of cysteine and glutathione were higher in the Pb-accumulating ecotypes of *Sedum alfredii* than those of the Pb non-accumulating ecotypes [24]. In the majority of cases, only a small part of the Pb absorbed by the roots can be transported to the shoots [24]. For the whole plant, the accumulation capacity of *M. cordata* for Zn is higher than that for Pb; for example, the Zn content in the shoots can reach to 1072 μg·g^−1^ (Figure 2b). Taking into consideration the large biomass of *M. cordata*, the Zn content of a single plant is more than some reported hyperaccumulators of Zn [25,26,27], so *M. cordata* can be considered as a Zn accumulator for phytoremediation [21]. Curiously, the Zn content of the roots and shoots of *M. cordata* was hardly affected by the Pb treatment, except when the Zn content significantly increased in the roots with 100 μmol·L^−1^ Pb (Figure 2c). Zn is an essential element; plants have many zinc transporters or zinc-binding proteins to control their Zn uptake and transport under heavy metal stress. This may be the reason why *M. cordata* roots have a high tolerance to excess Zn [28].

Large amounts of ROS are produced under heavy metal stress, which ultimately causes serious damage to membrane systems and proteins, and can even lead to the death of the plant [5,22,29]. The amount of H_2_O_2_ and O_2_^−^ production in plant tissues can be detected by histochemical staining of DAB and NBT, or measuring the H_2_O_2_ content and O_2_^−^-producing rate [10]. In the present study, the O_2_^−^ production had a significant increase in the roots of *M. cordata* with the Zn treatment; however, there was no significant change with the Pb treatment (Figure 3 and Figure 4), which was consistent with the activity change of NADPH oxidase under the Pb and Zn treatments (Figure 5). NADPH oxidases can use cytosolic NADPH to produce O_2_^−^, which is quickly transformed to H_2_O_2_ by SOD or spontaneous action [10,30]. Besides NADPH oxidase, pH-dependent cell wall peroxidases have been proposed as sources of H_2_O_2_ in the apoplast [31]. In a previous study, we also found that an increase in NADPH oxidase activity was involved in the ROS production of Cd-induced *Solanum nigrum* [11], Cu-induced *E. haichowensis* [10], and Zn-induced *S. alfredii* [13], and the increase in [Ca^2+^]_cyt_ and root hair development in the roots of *S. alfredii* [13]. The Pb-induced H_2_O_2_ increase in the roots of *M. cordata* was similar to that of the Zn-induced increase in the present study, except a small amount of damage was observed on the root tips with the 200 μmol·L^−1^ Zn treatment (Figure 3b), denoting the root tips of *M. cordata* were damaged to some extent by Zn, but not by the Pb treatment.

SOD, CAT, APX and POD constitute the important defense enzyme systems to various stresses in plants [11,32,33]. SOD mainly reduces toxicity by scavenging O_2_^−^. CAT and APX can scavenge excessive H_2_O_2_ to maintain normal levels in the cells of plant. POD can not only catalyze the oxidation reaction of H_2_O_2_, but can also catalyze the conversion of H_2_O_2_ and O_2_^−^ into hydroxyl radicals to intensify peroxidation [34]. In this study, the enzyme activities of SOD, CAT, APX and POD in the roots significantly increased under Pb stress compared with those of the control (Figure 6). Nonetheless, the antioxidant enzyme activities in the roots significantly decreased with the Zn treatment except for 50 μmol·L^−1^ Zn (Figure 7). The difference in antioxidant enzyme activity may be related to the damage of the roots of *M. cordata* under the Pb and Zn treatments. This finding showed that the O_2_^−^ produced by NADPH oxidase in the roots of *M. cordata* with excess Zn was not been eliminated owing to the decrease in antioxidant enzyme activity; however, the ROS induced by Pb can be removed by the antioxidant enzyme system in the roots of *M. cordata*. Zn-induced ROS were produced and antioxidant enzymes decreased in the roots of the hyperaccumulating ecotype of *S. alfredii* [13]. It was also reported that the translocation of heavy metals from the roots to shoots was a detoxification pathway to reduce the stress toxicity to the plant roots [4].

## 4. Materials and Methods

### 4.1. Hydroponic Culture

The seeds of *M. cordata* were collected from mine tailing of Huaguoshan in Luoyang, China. After the seeds were germinated in vermiculite, the seedlings with uniform growth were transferred to 2.5 L plastic vessels containing Hoagland nutrient solution (1 mM KH_2_PO_4_, 1 mM KNO_3_, 1 mM Ca(NO_3_)_2_, 1 mM MgSO_4_, 20 μM Fe-EDTA, 46 μM H_3_BO_3_, 9 μM MnCl_2_, 0.76 μM ZnSO_4_, 0.32 μM CuSO_4_, 0.11 μM H_2_MoO_4_) and were grown under controlled conditions (14 h day length with photosynthetically active radiation of 400 μmol m^−2^ s^−1^ and 25/20 °C day/night temperatures). The solution pH was adjusted to 5.3 with NaOH or HCl with renewal of the nutrient solution every 2 days. Seedlings were treated with varying concentrations of Pb and Zn for 7 days after four leaves had appeared. The control (CK) was cultivated in a complete Hoagland solution only (0.76 μmol·L^−^^1^ Zn and without the addition of Pb). The heavy metals were applied with 50, 100 and 200 μmol·L^−^^1^ ZnSO_4_·7H_2_O for the Zn treatment and 10, 50 and 100 μmol·L^−^^1^ Pb(NO_3_)_2_ for the Pb treatment, and each treatment had three replicate vessels. After 7 days of exposure, the roots and shoots were then removed and separated for metal content determination, and the fresh roots were removed for detection of in situ staining, H_2_O_2_ content, O_2_^−^-producing rate and enzymatic activity analysis.

### 4.2. Metal Content Analysis

Shoots and roots of *M. cordata* were collected after washing them with distilled water, and the whole roots were immersed in 25 mmol·L^−1^ EDTA-Na_2_ solution for 10 min and then washed with distilled water again. The samples were then oven-dried at 80 °C and weighed to obtain dry weight. About 0.2 g of the dried samples were digested by the mixed acid of HNO_3_:HClO_4_ (87:13, *v*/*v*) following the procedure described by Zhang et al. [21]. The ICP-OES (Optima 8000, PerkinElmer, Waltham, MA, USA) was used to analyze the content of Zn and Pb.

### 4.3. Histochemical Detection of Metals, H_2_O_2_ and O_2_^−^ In Situ

The H_2_O_2_ and O_2_^−^ formation in situ in roots was detected separately by DAB and NBT staining as reported by Zhang et al. [22]. The roots were immersed in 25 mmol·L^−1^ EDTA-Na_2_ solution for 10 min and then washed with distilled water before detection of metals in situ. Subsequently, the cut roots were immersed in a staining solution for 15 min. DAB staining solution: 1mg·mL^−1^ solution of DAB with a pH of 3.8. NBT staining solution: 0.1% NBT in 50 mM of K-phosphate buffer containing 10 mM sodium azide with a pH of 6.4. After thoroughly washing them with distilled water, the well-stained root tips were immediately observed under an Olympus microscope fitted with a Nikon D7100 digital camera.

### 4.4. Determination of H_2_O_2_, and O_2_^−^ in the Root Extracts

The content of H_2_O_2_ was measured by monitoring the absorbance at 415 nm of the titanium–peroxide complex following the method described by Jiang and Zhang [30]. The production rate of O_2_^−^ was measured by monitoring the absorbance at 530 nm of the nitrite formation from hydroxylamine hydrochloride in the presence of O_2_^−^, as described by Jiang and Zhang [30].

### 4.5. Isolation of the Plasma Membrane and Determination of NADPH Oxidase Activity

Plasma-membrane-enriched fractions were isolated as described by Zhang et al., with some modifications [10]. Briefly, the roots were homogenized in a pre-cooled mortar with two volumes of freshly prepared media containing 250 mM sucrose, 50 mM Tris-HCl buffer (pH 7.5), 5 mM EDTA, 1 mM phenylmethylsulfonyl fluoride (PMSF), and 1.5% (*w*/*v*) polyvinylpolypyrrolidone (PVPP). The homogenate was filtered through four layers of cheesecloth and then centrifuged at 12,000× *g* for 30 min at 4 °C, then the supernatant was centrifuged at 100,000× *g* for 40 min at 4 °C to obtain a microsomal membrane pellet. The pellet was gently resuspended in medium with 5 mM phosphate buffer (pH 7.8) containing 0.33 mM sucrose and 2 mM dithiothreitol (DTT), and isolated by an aqueous two-phase polymer system to give a final composition of 6.2% (*w*/*w*) dextran T500, 6.2% (*w*/*w*) PEG 3350, 0.33 M sucrose, 3 mM KCl, and 5 mM phosphate buffer (pH 7.8). Three successive rounds of partitioning followed. The upper phase was diluted five-fold in Tris-HCl buffer (10 mM, pH 7.5) containing 0.25 M sucrose, 1 mM EDTA, 1 mM DTT and 1 mM PMSF. All procedures were carried out at 4 ◦C. The plasma membrane pellets were quickly frozen and stored at −80 °C until they were used for the enzyme assays. To check the degree of enrichment of the plasma membrane, vanadate-sensitive (100 mM vanadate), nitrate-sensitive (100 mM KNO_3_) and azide-sensitive (1 mM NaN_3_) ATPase activities (plasma membrane, tonoplast, and mitochondrial enzyme markers, respectively) were determined in plasma membrane pellets as described by Zhang et al. [35].

The plasma-membrane NADPH oxidase activity was assayed by measuring NADPH-dependent reduction of sodium 3′-[1-[phenylamino-carbonyl]-3,4-tetrazolium]-bis(4-methoxy-6-nitro) benzenesulfonic acid hydrate (XTT) by O_2_^−^. The procedure was performed according to the methods of Sagi and Fluhr [36].

### 4.6. Enzyme Activity Assay

Fresh roots were extracted with precooling extraction buffer (including 50 mM potassium phosphate with 1 mM EDTA and 1% PVPP (*w*/*v*) with a pH of 7.0) on the ice, as described by Zhang et al. [10]. CAT activity was determined by measuring the consumption of H_2_O_2_ at 240 nm according to the method of Aebi [37]. APX activity was measured by monitoring the decrease in the absorbance at 290 nm as ascorbate was oxidized, as described by Nakano and Asada [38]. POD activity was determined by measuring the change in the absorption at 470 nm according to Zheng and Van Huystee [39]. SOD activity was determined by its ability to inhibit the formation of nitroblueformazan from NBT according to the method of Giannopolitis and Ries [40]. Protein content was estimated with the same root extracts according to Bradford [41].

### 4.7. Statistical Analysis

Data were analyzed using one-way variance of SPSS 22.0. Values were presented as means ± SE (*n* = 3) with three separate replicates, and means denoted by different letters refer to the significant differences (*p* < 0.05, Duncan’s test). The staining experiments were repeated at least five times with similar results.

## 5. Conclusions

In this study, Pb was mainly accumulated in the roots of *M. cordata* with the Pb treatment, and less than 1% Pb was transported to the parts above; however, our analysis of the Zn content demonstrated a 39% accumulation in the shoots. The high tolerance of *M. cordata* to Pb can be realized by fixing the Pb in the apoplast of the roots and limiting its transport to the aboveground parts. Moreover, the Pb induced the H_2_O_2_ production in the roots, and a significant increase in the activities of the antioxidant enzymes, which further improved the Pb tolerance of the roots of *M. cordata*. On the other hand, the Zn stress induced large amounts of H_2_O_2_ and O_2_^−^ in the roots, but was accompanied by a significant decrease in the activities of antioxidant enzymes, which caused oxidative damage to the root tips. In order to reduce the root damage, the excess Zn in the roots of *M. cordata* can be transported to the shoots. The different mechanisms of tolerance to the Pb and Zn in the roots of *M. cordata* may be beneficial as an excellent remediation material for soil polluted with Pb–Zn-compounds. Pb–Zn-contaminated soil as a culture medium will be tested in the future to support the conclusions of the study.

## Data Availability

Not applicable.

## References

[1] Ali H., Khan E., Sajad M.A. (2013). Phytoremediation of heavy metals-Concepts and applications. Chemosphere.

[2] Wan X., Lei M., Chen T. (2016). Cost–benefit calculation of phytoremediation technology for heavy-metal-contaminated soil. Sci. Total Environ..

[3] Bolan N.S., Jin H.P., Robinson B., Naidu R., Huh K.Y. (2011). Phytostabilization: A green approach to contaminant containment. Adv. Agron..

[4] Tang Z., Wang H., Chen J., Chang J., Zhao F. (2022). Molecular mechanisms underlying the toxicity and detoxification of trace metals and metalloids in plants. J. Integr. Plant Biol..

[5] Clemens S. (2006). Toxic metal accumulation, responses to exposure and mechanisms of tolerance in plants. Biochimie.

[6] Baker A.J.M., Reeves R.D., Hajar A.S.M. (1994). Heavy metal accumulation and tolerance in British populations of the metallophyte *Thlaspi caerulescens* J & C Presl. (Brasicaceae). New Phytol..

[7] Kamran M., Asif S., Nadeem H., Saddam S., Muhammad R. (2017). Phytoremediation strategies for soils contaminated with heavy metals: Modifications and future perspectives. Chemosphere.

[8] Fukao Y., Ferjani A., Tomioka R., Nagasaki N., Kurata R., Nishimori Y., Maeshima F.M. (2011). iTRAQ analysis reveals mechanisms of growth defects due to excess zinc in Arabidopsis. Plant Physiol..

[9] Reeves R.D., Baker A.G.M., Terry N., Banuelos G.S. (2000). Metal accumulation plant. Phytoremediation of Toxic Metals: Using Plant to Clean up the Environment.

[10] Zhang H., Zhang F., Xia Y., Wang G., Shen Z. (2010). Excess copper induces production of hydrogen peroxide in the leaf of *Elsholtzia haichowensis* through apoplastic and symplastic CuZn-superoxide dismutase. J. Hazard. Mater..

[11] Deng X., Xia Y., Hu W., Zhang H., Shen Z.G. (2010). Cadmium-induced oxidative damage and protective effects of N-acetyl-L-cysteine against cadmium toxicity in *Solanum nigrum* L.. J. Hazard. Mater..

[12] Zhang X., Zhang H., Lou X., Tang M. (2019). Mycorrhizal and non-mycorrhizal *Medicago truncatula* roots exhibit differentially regulated NADPH oxidase and antioxidant response under Pb stress. Environ. Exp. Bot..

[13] Jin X., Yang X., Islam E., Liu D., Mahmood Q., Li H., Li J. (2008). Ultrastructural changes, zinc hyperaccumulation and its relation with antioxidants in two ecotypes of *Sedum alfredii* Hance. Plant Physiol. Biochem..

[14] Sai C., Li D., Xue C., Wang K., Hu P., Pei Y., Bai J., Jing Y., Li Z., Hua H. (2015). Two pairs of enantiomeric alkaloid dimers from *Macleaya cordata*. Org. Lett..

[15] Liu X., Liu Y., Huang P., Ma Y., Qing Z., Tang Q., Cao H., Cheng P., Zheng Y., Yuan Z. (2017). The genome of medicinal plant *Macleaya cordata* provides new insights into benzylisoquinoline alkaloids metabolism. Mol. Plant.

[16] Li C., Hu N., Ding D., Hu J., Li G., Wang Y. (2015). Phytoextraction of uranium from contaminated soil by *Macleaya cordata* before and after application of EDDS and CA. Environ. Sci. Pollut. Res..

[17] Wang J., Wang X., Li J., Zhang H., Xia Y., Chen C., Shen Z., Chen Y. (2018). Several newly discovered Mo-enriched plants with a focus on *Macleaya cordata*. Environ. Sci. Pollut. Res..

[18] Nie J., Liu Y., Zeng G., Zheng B., Tan X., Liu H., Xie J., Gan C., Liu W. (2016). Cadmium accumulation and tolerance of *Macleaya cordata*: A newly potential plant for sustainable phytoremediation in Cd-contaminated soil. Environ. Sci. Pollut. Res..

[19] Cai B., Chen Y., Du L., Liu Z., He L. (2021). Spent mushroom compost and calcium carbonate modification enhances phytoremediation potential of *Macleaya cordata* to lead-zinc mine tailings. J. Environ. Manag..

[20] Pan G., Zhang H., Liu W., Liu P. (2019). Integrative study of subcellular distribution, chemical forms, and physiological responses for understanding manganese tolerance in the herb *Macleaya cordata* (papaveraceae). Ecotoxicol. Environ. Saf..

[21] Zhang H., Zhou W., Chen Y., Xu H., Hou D., Lv S., Sun X., Wang F., Yang L. (2022). The tolerance, absorption, and transport characteristics of *Macleaya cordata* in relation to lead, zinc, cadmium, and copper under hydroponic conditions. Appl. Sci..

[22] Zhang H., Xia Y., Wang G., Shen Z. (2008). Excess copper induces accumulation of hydrogen peroxide and increases lipid peroxidation and total activity of copper-zinc superoxide dismutase in roots of *Elsholtzia haichowensis*. Planta.

[23] Enot M.M., Weiland F., Mittal P., Hoffmann P., Sillero-Mahinay M., Pukala T. (2021). Differential proteome analysis of the leaves of lead hyperaccumulator, *Rhoeo discolor* (L. Her.) Hance. J. Mass. Spectrom..

[24] Gupta D.K., Huang H.G., Yang X.E., Razafindrabe B., Inouhe M. (2010). The detoxification of lead in *Sedum alfredii* H. is not related to phytochelatins but the glutathione. J. Hazard. Mater..

[25] Meyer-Klaucke W., Kupper H., Mijovilovich A., Pmh K. (2004). Tissue- and age-dependent differences in the complexation of cadmium and zinc in the cadmium/zinc hyperaccumulator *Thlaspi caerulescens* (Ganges ecotype) revealed by X-ray absorption spectroscopy. Plant Physiol..

[26] Vázquez M., Poschenrieder C., Barceló J., Baker A., And P.H., Cope G.H. (1994). Compartmentation of zinc in roots and leaves of the zinc hyperaccumulator *Thlaspi caerulescens* J & C Presl. Bot. Acta.

[27] Sarret G., Saumitou-Laprade P., Bert V., Proux O., Hazemann J.L., Traverse A., Marcus M., Manceau A. (2003). Forms of zinc accumulated in the hyperaccumulator *Arabidopsis halleri*. Plant Physiol..

[28] Shinozaki D., Yoshimoto K. (2021). Autophagy balances the zinc-iron seesaw caused by Zn-stress. Trends Plant Sci..

[29] Romero-Puertas M., Rodriguez-Serrano M., Corpas F., Gomez M., Rio L., Sandalio L. (2004). Cadmium-induced subcellular accumulation of O_2_^·−^ and H_2_O_2_ in pea leaves. Plant Cell Environ..

[30] Jiang M., Zhang J. (2001). Effect of abscisic acid on active oxygen species, antioxidative defence system and oxidative damage in leaves of maize seedlings. Plant Cell Physiol..

[31] Quan L., Zhang B., Shi W., Li H. (2008). Hydrogen peroxide in plants: A versatile molecule of reactive oxygen species network. J. Integr. Plant Biol..

[32] Zhang H., Lv S., Xu H., Hou D., Li Y., Wang F. (2017). H_2_O_2_ is involved in the metallothionein-mediated rice tolerance to copper and cadmium toxicity. Int. J. Mol. Sci..

[33] Zhang H., Xia Y., Chen C., Zhuang K., Song Y., Shen Z. (2016). Analysis of copper-binding proteins in rice radicles exposed to excess copper and hydrogen peroxide stress. Front. Plant Sci..

[34] Sgherri C., Quartacci M.F., Navari-Izzo F. (2007). Early production of activated oxygen species in root apoplast of wheat following copper excess. J. Plant Physiol..

[35] Zhang F., Zhang H., Wang G., Xu L., Shen Z. (2009). Cadmium-induced accumulation of hydrogen peroxide in the leaf apoplast of *Phaseolus aureus* and *Vicia sativa* and the roles of different antioxidant enzymes. J. Hazard. Mater..

[36] Sagi M., Fluhr R. (2001). Superoxide production by plant homologues of the gp91phox NADPH oxidase. Modulation of activity by calcium and by tobacco mosaic virus infection. Plant Physiol..

[37] Aebi H. (1984). Catalase in vitro. Method. Enzymol..

[38] Nakano Y., Asada K. (1980). Hydrogen peroxide is scavenged by ascorbate-specific peroxidase in Spinach chloroplasts. Plant Cell Physiol..

[39] Zheng X., Huystee R. (1992). Peroxidase-regulated elongation of segments from peanut hypocotyls. Plant Sci..

[40] Giannopolitis C., Ries S. (1977). Superoxide dismutases: I. Occurrence in higher plants. Plant Physiol..

[41] Bradford M.M. (1976). A rapid and sensitive method for the quantification of microgram quantities of protein utilizing the principle of protein dye-binding. Anal. Biochem..

